# Long Non-Coding RNAs, Extracellular Vesicles and Inflammation in Alzheimer’s Disease

**DOI:** 10.3390/ijms232113171

**Published:** 2022-10-29

**Authors:** Ania Canseco-Rodriguez, Valeria Masola, Vincenza Aliperti, Maria Meseguer-Beltran, Aldo Donizetti, Ana María Sanchez-Perez

**Affiliations:** 1 Neurobiotecnology Group, Faculty of Health Science, Institute of Advanced Materials (INAM), University of Jaume I, 12006 Castellon, Spain; 2 Department of Biology, University of Naples Federico II, 80126 Napoli, Italy

**Keywords:** Alzheimer’s disease, inflammation, non-coding RNAs, exosome vesicles

## Abstract

Alzheimer’s Disease (AD) has currently no effective treatment; however, preventive measures have the potential to reduce AD risk. Thus, accurate and early prediction of risk is an important strategy to alleviate the AD burden. Neuroinflammation is a major factor prompting the onset of the disease. Inflammation exerts its toxic effect via multiple mechanisms. Amongst others, it is affecting gene expression via modulation of non-coding RNAs (ncRNAs), such as miRNAs. Recent evidence supports that inflammation can also affect long non-coding RNA (lncRNA) expression. While the association between miRNAs and inflammation in AD has been studied, the role of lncRNAs in neurodegenerative diseases has been less explored. In this review, we focus on lncRNAs and inflammation in the context of AD. Furthermore, since plasma-isolated extracellular vesicles (EVs) are increasingly recognized as an effective monitoring strategy for brain pathologies, we have focused on the studies reporting dysregulated lncRNAs in EVs isolated from AD patients and controls. The revised literature shows a positive association between pro-inflammatory lncRNAs and AD. However, the reports evaluating lncRNA alterations in EVs isolated from the plasma of patients and controls, although still limited, confirm the value of specific lncRNAs associated with AD as reliable biomarkers. This is an emerging field that will open new avenues to improve risk prediction and patient stratification, and may lead to the discovery of potential novel therapeutic targets for AD.

## 1. Alzheimer’s Disease and Inflammation

Acute inflammation, as part of the innate immune system, plays a protective role when injury or damage occurs. However, the chronic inflammatory response has the opposite effect, existing as a decisive mechanism underlying several human pathologies [1]. Neuroinflammation is characterized by the hyperactivation of microglia (the brain resident macrophages) and astrocytes. Accumulated evidence indicates that neuroinflammation in different brain areas is a common denominator in several degenerative disorders of various etiology, e.g., Huntington’s disease [2], Parkinson’s disease (PD) [3], and Alzheimer’s disease (AD) [4,5]. In AD, the association of amyloid plaques (Aβ) deposits and tau neurofibrillary tangles (NFT) with neuroinflammation has been extensively acknowledged [6,7,8,9,10] to the point that immunotherapies have been proposed to ameliorate AD [11]. Aβ oligomers, among other harmful stimuli, accumulate through a lifetime, and can continuously stimulate microglial cells [12,13,14]. The sustained activation of the immune response leads to the chronic production of pro-inflammatory cytokines that, in turn, are toxic to neurons. Damaged neurons undergo different pathological processes that lead to an increment of amyloid precursor protein and increased Aβ secretion, thus worsening the inflammatory response [15,16,17,18]. This negative cycle leads to impaired brain function and, eventually, massive neuronal death. Targeting neuroinflammation has been proposed in several clinical trials to prevent the progression of the disease (reviewed in [19]).

## 2. Non-Coding RNAs

Most of the human genome is transcribed into non-coding RNAs (ncRNAs). ncRNAs are classified into structural and regulatory ncRNAs. The first class includes ribosomal RNAs (rRNAs), transfer RNAs (tRNAs), small nuclear RNAs (snRNAs), and small nucleolar RNAs (snoRNAs). The second group is further classified into different categories according to their size: short ncRNAs, including microRNAs (miRNAs, 22–23 nucleotides, nts) and piwiRNAs (piRNAs, 26–31 nts); medium ncRNAs (50–200 nts); and long ncRNAs (lncRNAs, >200 nts) [20,21,22]. ncRNAs can regulate gene expression either positively or negatively through different molecular mechanisms and at different levels, from chromatin remodeling to mRNA translation [23,24,25,26]. Moreover, different ncRNAs can interact with each other in an intricate network to regulate their stability and abundance [27]. ncRNAs are highly expressed in the brain, and their expression pattern is finely regulated [28,29]. Brain function and development are widely affected by ncRNA action [21,30], thus dysregulation of ncRNA expression and/or function may lead to brain disorders ranging from neurological and neuropsychiatric diseases to tumorigenesis [31,32].

### Long Non-Coding RNAs

LncRNAs are typically transcribed by Polymerase II, and their structure resembles mRNAs since they are 5′-capped, polyadenylated, and spliced [32,33]. However, unlike mRNAs, lncRNAs are shorter, less stable, and show lower expression levels with a tissue-specific expression pattern [32].

LncRNAs express during development, playing a key role in the regulation of a wide range of cellular processes, acting both as chromatin regulators and by regulating gene expression at the transcriptional and post-transcriptional levels [34]. In addition, lncRNAs fold into thermodynamically stable secondary structures characterized by different functional domains (DNA-binding domains, RNA-binding domains, protein-binding domains, and conformational switches) [35]. In this way, lncRNAs can physically and functionally interact with the other biomolecules (DNA, RNA, and proteins) both by base-pairing with complementary nucleic acid sequences or through these functional domains [36]. They can be classified into several major categories based on their position relative to neighboring protein-coding genes (sense, antisense, bidirectional, intergenic transcripts), their subcellular localization, and their mechanism of action (*cis*- or *trans*-acting lncRNAs) [22,32]. *Cis*-acting lncRNAs influence the expression of nearby genes located in the same chromosome as their own sites of transcription [37], in contrast with *trans*-acting lncRNAs, which can operate in distant regions in different chromosomes [26].

## 3. Long Non-Coding RNAs in Alzheimer’s Disease Related to Inflammation

The dysregulation of ncRNAs associated with AD is well acknowledged [38,39] and lncRNAs in association with AD have been extensively reviewed [40,41,42,43]. Some studies focus on the role of lncRNAs as potential AD biomarkers [44], while others focus on lncRNAs showing competing endogenous RNA network (ceRNA) mechanisms [45]. More recently, a group of lncRNAs has been suggested as potential therapeutic targets for AD [46].

Since neuroinflammation is a central mechanism of AD, attention has focused on the possible immune-modulatory activities of lncRNAs, revealing that they can positively and/or negatively regulate innate immune gene expression through their general mechanisms of action (miRNA sponge, chromatin remodeling, transcriptional activation/inhibition, post-transcriptional modification) and even regulation of protein activity [47,48,49]. Transcriptomic studies have highlighted the role of different lncRNA-associated ceRNA networks in the overexpressing APP/PS1 mice model, associated with an early stage of AD, mainly involved in synaptic plasticity, memory, and neuroinflammation [50,51]. In the brain, inflammatory stimuli, such as the one caused by lipopolysaccharides (LPS), regulate the expression of genes via the upregulation of several lncRNAs [52]. Furthermore, the lncRNA modulation of neuroinflammation is increasingly acknowledged as a key mechanism underlying nervous system disorders [53].

A recent review, while we were in the revision process, has highlighted the role of non-coding RNA, in regulating inflammation in AD [54]. In that manuscript, only proinflammatory lncRNA is described. Here, we detailed the updated literature that, experimentally, has demonstrated the association of specific lncRNAs with inflammatory processes in the context of AD. According to their reported effect in cellular, animal models, and patients, we classify lncRNAs as pro-inflammatory or anti-inflammatory (Table 1).

### 3.1. Pro-Inflammatory lncRNAs, Evidence from Cellular, Animal Models, and Human Studies

To date, the majority of reported neuroinflammation-associated lncRNAs regarding AD progression have a pro-inflammatory role.

LncRNA *Antisense Non-coding RNA in the INK4 locus* (ANRIL) is mapped at the INK4 (Inhibitor of Cyclin-dependent Kinase 4) locus and has been identified in several diseases that are related to inflammation and nerve dysfunction [86]. In pheochromocytoma cells (PC12), a well-known neuronal cellular model, lncANRIL was upregulated by incubating the cells with Aβ(1–42) oligos. Moreover, the lncANRIL knockdown decreased inflammatory cytokine expression, inhibited Aβ-induced apoptosis and autophagy, and led to increased neurite outgrowth, by binding and downregulating miR-125a [56]. In models of coronary disease, lncANRIL can increase NFkB expression via miR-181b modulation [57], but whether miR-181b is targeted in neuronal cells is still unknown. Moreover, whether these changes are reflected in human or animal models of AD remains elusive.

LncRNA *BACE 1 Antisense RNA* (BACE1-AS), the antisense of BACE1 (β-secretase), improves BACE1 mRNA stability, preventing the binding of miR-485-5p, thus increasing BACE1 levels [59]. In a neuronal cellular model of PD, BACE1-AS can regulate apoptosis, inflammatory response, and oxidative stress, through direct regulation of the miR-214-3p/CDIP1 (Cell Death Inducing P53 Target 1) signaling axis [60].

Interestingly, BACE1-AS/BACE1 dysfunction underlies several human diseases with strong inflammatory components, including multiple tumors and degenerative diseases [87]. BACE1-AS is upregulated in serum samples of AD patients and brain tissues of AD transgenic (Tg) mice [58], promoting neuronal damage mediated by autophagy by binding to miR-214-3p and indirectly inhibiting ATG5 expression [58].

LncRNA *Brain-derived Neurotrophic Factor Antisense* (BDNF-AS) has been reported as a target of anti-inflammatory treatments. Specifically, lithium treatment decreased inflammation via decreasing BDNF-AS levels and increasing its target miR-9-5p in a rat model of spinal cord injury (SCI), and it reduced the inflammatory effect caused by H_2_O_2_ in SH-SY5Y cells [61]. According to this putative role in facilitating inflammation, increased levels of BDNF-AS can impair cognition in neurodegenerative preclinical models. Moreover, elevated levels of BDNF-AS are found in AD patients’ blood [62]. Despite this evidence, the direct role of BDNF-AS on inflammation in AD models has not yet been reported.

LncRNA *HOX Antisense Intergenic RNA* (HOTAIR) is highly expressed in inflammatory conditions, e.g., tumors, traumatic brain injury mice model, and LPS-treated microglial (BV2) cells. Accordingly, silencing HOTAIR suppresses microglial activation and the release of inflammatory factors [53]. Supporting a pro-inflammatory role of HOTAIR, sulfasalazine (used for the treatment of autoimmune diseases) reduces HOTAIR expression and prevents the increment of M1-like microglia in a mice model of cuprizone-induced demyelination [63]. Furthermore, exercise downregulates HOTAIR, and increases its target miR-130a-3p in rat models of AD. In this study, treadmill exercise exerts neuroprotection by reducing inflammatory microglia and oxidative stress, and consequently, improving cognitive function [64]. In humans, aerobic exercise can attenuate the white matter hyperintensities associated with AD and aging [88]. Not surprisingly, exercise has been proposed as a useful strategy to prevent AD [89], due to its potential anti-inflammatory effect [90]. However, whether the anti-inflammatory effect of exercise is mostly mediated by HOTAIR reduction, or whether this is a downstream event, has not been demonstrated.

LncRNA 17A is a 159-nts antisense transcript, embedded in the human G-protein-coupled receptor 51 gene (GPR51), GABA B2 receptor. The stable expression of 17A in SH-SY5Y cells promotes an alternative GABA B splicing isoform that inhibits GABA B intracellular signaling [55]. Synthesis of 17A is controlled by inflammatory processes, and it is upregulated in the cerebral cortex of AD patients and appears to enhance the secretion of Aβ in SH-SY5Y cells as a response to inflammatory stimuli [55]. Impaired GABAergic function plays a significant role in AD, as alterations in this kind of transport account for neurodegenerative diseases, specifically the shift of GABA to depolarizing direction because of the impairment of the KCC2 (*potassium chloride cotransporter 2*). In AD11 mice, a model of sporadic AD, the neutralization of NGF (*nerve growth factor*) leads to a neurodegenerative pathology such as the one observed in AD patients [91]; thus, this lncRNA represents an interesting link between inflammation and AD.

LncRNA *Membrane-associated Guanylate Kinase Antisense RNA 3* (MAGI2-AS3) Aβ_25–35_ incubation of SH-SY5Y and BV2 leads to increased expression of lncMAGI2-AS3 that results in reduced levels of its target miR-374b-5p levels. This result suggests that MAGI2-AS3/miR-374b-5p axis may regulate the neurotoxicity and neuroinflammation induced by Aβ_25–35_ [92]. Furthermore, miR-374b-5p appears to be important in neurogenesis and it is found downregulated in AD patients, coherent with a pro-inflammatory role of MAGI2-AS3.

In that sense, and although no studies on MAGI2-AS3 in AD patients have been reported yet, MAGI2-AS3 appears in a screening of the ceRNA network in human asthma studies [93], confirming a potential pro-inflammatory role of MAGI2-AS3.

LncRNA N336694 is found up-regulated in APP/PS1 mice brain tissue, suggesting a pro-inflammatory role [94]. In this study, miR-1066 was also found upregulated, and, although bioinformatic analysis suggested that miR-1066 may be a potential target of lncRNA n3366994, no empirical confirmation has been reported. Interestingly, simvastatin treatment that ameliorated cognition in mice models of AD, was shown to suppress lncRNA n3366994 and miR-106b expression in the brain in APP/PS1 mice [65].

LncRNA *Neuroblastoma differentiation marker* 29 (NDM29) is a lncRNA transcribed by RNA pol III, embedded in the first intron of the ASCL3 (*achaete scute-like homolog* 3) gene in humans. NDM29 expression is enhanced in the cerebral cortex of AD patients, its biosynthesis responds to pro-inflammatory molecules, and it is downregulated by anti-inflammatory drugs in different neuroblastoma cell lines [66].

*Nuclear paraspeckles assembly transcript* 1 (NEAT1) is upregulated in the temporal cortex and hippocampus of AD patients compared to controls [69]. NEAT1 can modulate inflammatory processes in several cell types and several human pathologies of inflammatory conditions, via the modulation of several miRNAs, in the AKT, TLR4, TRAF6, and NF-κB signaling pathways [68]. In SH-SY5Y cells, Aβ protein incubation increased NEAT1 and decreased its target miR27a-3p [67].

LncRNA *Prostate androgen-regulated transcript* 1 (PART1) has a key role in a variety of biological processes. Using Aβ_1–42_-incubated endothelial cells as a model of the blood-brain barrier (BBB), lncRNA PART1 increased BBB permeability via binding NOVA2. Thus, in this model, high expression of lncPART1 led to reduced PPP2R3A mRNA levels and subsequently increased NFkB-p65 phosphorylation. NOVA2 (*neuro-oncological ventral antigen 2*) expression is reduced in this environment and stabilizes lncPART1, resulting in sustained NFkB-p65 phosphorylation. This signaling contributes to the alteration of BBB proteins (e.g., occludin, claudin-5, and ZO-1), leading to increased permeability. Although this could be a potential mechanism aggravating AD pathogenesis, more studies are needed to unravel the function of this lncRNA in AD, as a diagnostic and therapeutic [70].

LncRNA *Ribonuclease P RNA component* H1 (Rpph1) participates in the maturation of tRNA [95]. This lncRNA is upregulated in the cortex of an APPswe/PS1deltaE9 mice model of Alzheimer’s, compared to wild-type controls. In this model, Rpph1 upregulates CDC42 (*Cell division cycle 42*) regulation, by competing with miR-330-5p and miR326-3p. CDC42 modulates actin dynamics, promoting dendritic spine formation [84,95]. Furthermore, Rpph1 displays a neuroprotector effect in SH-SY5Y cells incubated with Aβ via miR326-3p/PKM2 (pyruvate kinase isoform M2) axis [85]. However, Rpph1 overexpression can promote inflammation under low glucose conditions in a model of mesangial cells [83]; thus, it is still unclear whether the increased levels in the AD mice model are due to early compensation or to a pro-inflammatory role. At this point, the exact function of Rpph1 in AD remains elusive.

LncRNA *SOX21-antisense Transcript* 1 (SOX21-AS1) represses the expression of the SOX21 gene, a member of the large SOX (*SRY-related HMG-box genes*) family of transcription factors involved in development regulation [96]. It is mostly studied for its oncogenic properties. In AD mice, knocking down SOX21-AS prevents neuronal oxidative stress and inhibits cell death, through the upregulation of the FZD3–5 (*Frizzled receptor* 3)/Wnt signaling pathway [77] and given the close relationship between oxidative stress and inflammation [97], SOX21-AS1 can be classified as pro-inflammatory lncRNA.

LncRNA *SRY-box transcription factor 2 overlapping transcript* (SOX2-OT) is transcribed from the intron of the Sox2 gene [98] with a key role in maintaining SOX2 expression [99]. SOX2-OT is involved in neural embryonic development and adult mouse neurogenesis. Although adult neurogenesis is impaired in AD mice models [100], it is not known whether SOX2-OT dysfunction may contribute to the progress of the disease. However, SOX2-OT has been shown to mediate inflammation, oxidative stress, and neuronal apoptosis in PD cellular models, acting via miR-942-5p/NAIF1 (*Nuclear apoptosis-inducing factor* 1) axis [76]. Although there is no experimental evidence in AD cellular or animal models, a Logic Mining method used for the analysis of a microarray expression dataset shows SOX2-OT as one of the five genes common to both early and late AD states of the *anti-NGF AD11* transgenic mouse model, a model of sporadic AD [100]. Further studies are required to validate this gene in human transcriptional studies.

LncRNA *Small nucleolar RNA host gene* 1 (SNHG1) is a lncRNA that belongs to the Small Nucleolar RNA host gene (SNHG) family, comprising more than 20 members, many of which have been found associated with cancer progression [101]. In SH-SY5Y and human primary neuron cells, Aβ incubation increased the expression of SNHG1, while the silencing of this lncRNA attenuated Aβ-induced cellular death and alterations in mitochondrial membrane potential. In this study, SNHG1 was shown to act as a miR-137 sponge targeting KREMEN1 (*Kringles* Containing Transmembrane Protein 1) [73]. KREMEN1 is a transmembrane receptor that blocks the WNT/catenin pathway but can induce apoptosis independently [102]. Interestingly, silencing of KREMEN1 (by miR-431 overexpression) prevented Aβ-mediated synapse loss in primary cultures from a mice model of AD, suggesting that KREMEN1 may facilitate AD progression [103]. Another pathway regulated by SNHG1 is the miR-361-3p/ZNF217 axis in neuroblastoma cell lines (SK-N-SH and CHP 92 212); Aβ_25–35_ increased SNHG1 and reduced miR-361-3p, increasing its target ZNF217 (*zinc finger* gene 217 transcription factor) levels. ZNF217 is also the target of miR-212-3p [72] and miR-200 [71] in the context of Aβ_25–35_-induced inflammation in PC12 cells, where ZNF217 upregulation is associated with increased neurotoxicity.

LncRNA *Small Nucleolar RNA Host Gene* 14 (SNHG14) is another member of the SNHG family and has an essential role in promoting pro-inflammatory microglia activation [74]. In astrocytes from the transgenic APP/PS1 mice model, SNHG14 was reported to sponge miR-223-3p, which directly targets and restrains NLRP3 inflammasome. In this model, angiotensin analogs inhibit inflammation and prevent cognitive impairment by inhibiting SNHG14, thus restoring miR-223-3p function [74]. Exercise that improves cognition and reduces inflammation markers can also reduce SNG14 levels, in mice models and AD patients [75].

### 3.2. Anti-Inflammatory lncRNAs

Although less studied than the pro-inflammatory, some lncRNAs have an anti-inflammatory action in AD.

LncRNA *Maternally Expressed Gen* 3 (MEG3) expression declined in the hippocampus of AD model rats, and over-expressing MEG3 inhibited the activation of the astrocytes, reducing neuronal damage via the PI3K (Phosphoinositide 3 kinase)/AKT pathway [82]. No data on humans have been reported yet.

LincRNA-p21 was found upregulated by *Bilobalide* (the effective component of EGb76, extract of Ginkgo biloba), decreasing neuroinflammation, and promoting autophagy in a mice model of AD [78].

Another example is represented by MALAT1 (*Metastasis-associated Lung Adenocarcinoma Transcript* 1), which is downregulated in the cerebral-spinal fluid (CSF) in AD patients compared to controls [80,81]. MALAT1 levels correlate positively with alleviated AD severity, as evaluated by the Mini-mental Status examination (MMSE) score, and biomarkers Aβ_42_, t-tau, and p-tau. MALAT1 reduction and miR125b increase correlate with AD, but not with PD; suggesting that lncMALAT1/miR-125b are potential biomarkers for AD diagnosis. Consistently, in two cellular models of AD, MALAT1 was found to inhibit inflammation by sponging miR-125b [80]. Intriguingly, MALAT1 can promote neuroinflammation by NRF2 inhibition in a Parkinson’s Disease (PD) mouse model [79]. Further research is required to unveil the putative opposite role of MALAT1 in the inflammatory process underlying PD and AD.

## 4. Extracellular Vesicles in Neurodegenerative Diseases, a Link between lncRNAs, Inflammation, and AD

Extracellular vesicles (EVs) are heterogeneous membranous structures released into the extracellular space by all cell types. The main function of EVs is the intercellular communication underlying various physiological, but also pathological processes [104].

EVs are generally classified into three groups according to their origin, content, and size [105]. Apoptotic bodies are the largest size vesicles (around 5000 nm in diameter) and are released during apoptosis; microvesicles, the middle size group (100–1000 nm in diameter), are formed by plasma membrane outward invaginations; the smallest size vesicles, exosomes (30–150 nm in diameter), are released from the endosome system forming multivesicular bodies inside the cells before release.

EVs are characterized by a lipidic bilayer that contains cell organelles, DNA, cytosolic and membrane proteins, coding transcripts, and ncRNAs [106,107], including lncRNAs [108]. Once released into the interstitial spaces and body fluids, the secreted vesicles can be internalized by other cells, acting both on self and neighboring cells (autocrine and paracrine communication) and over long distances (endocrine communication) [109,110]. EVs can be internalized by recipient cells following receptor-ligand interactions without undergoing any structural and functional changes [111,112], and their cargos function as effector molecules in recipient cells [113]. EVs provide intercellular communication through the transport of different biological molecules. This way, EVs can also contribute to the spread of pathogenic agents.

All cell types in the central nervous system (CNS) (neurons, astrocytes, oligodendrocytes, microglia, and embryonic neural stem cells) can release EVs [114]. Their cargos are involved in the regulation of various biological processes, such as cell proliferation, differentiation [115,116], and synaptic plasticity [117], facilitating communication within the CNS. Currently, neuron to neuron, neuron to astrocyte, astrocyte to neuron, microglia to neuron, and oligodendrocyte to microglia and neuron, have been reported and described in [118]. In addition, between the CNS with other systems [115]. In turn, different external and internal stimuli will regulate EVs’ cargo, as this reflects the cellular status. Thus, in pathological conditions such as chronic inflammation, EVs will also undergo significant changes in their quantity, size, and cargo composition, thus reflecting the inflammatory condition of the cell of origin [119]. The mechanisms governing specific EVs cargo loading are complex and differ from MV and exosomes [118].

EVs have been generally considered in two types of applications: (i) the identification of pathology-specific molecules within EVs, demonstrating the potential for early diagnosis of diseases; (ii) as potential carriers of medicaments, given the low immunoreactivity, high biosafety, and the capability of specifically targeted delivery [120]. Furthermore, they could be used for the treatment of central nervous system disorders given their capability to cross the blood-brain barrier.

### Dysregulated Extracellular Vesicular lncRNAs in AD

Since EVs cargo reflects cellular status, the discovery of neuronal-derived EVs in the blood and CSF revolutionized the field of brain biomarkers, providing a non-invasive strategy that allows the evaluation of brain physio-pathological state. However, currently, the selective, reliable, and high-yield isolation of EVs from blood is still a challenging task, and there are no standardized methods for specific EVs isolation [118,121].

As a good reflection of cellular status, the ncRNA profiles contained in plasma EVs (particularly, plasma membrane-derived microvesicles (MVs) and endosome-derived exosomes) are altered in pathological situations, e.g., cancer, metabolic, and cardiovascular diseases [122]. Similarly, in neuronal-derived EVs isolated from AD patients, altered expression profiles of several miRNAs have been found [123,124]. However, to date, few studies have characterized lncRNAs in EVs directly involved in AD. Those studies include lncRNA BACE1-AS, which is found up-regulated in exosomes isolated from the plasma of AD patients [125]. PCA3 and RP11-462G22.1 are also up-regulated in EVs isolated from the CSF of AD patients [126]. PCA3 is known for its involvement in prostate cancer [127], whereas RP11-462G22.1 was originally found as a muscular dystrophy-associated lncRNA [128]. However, their biological function in AD is still unknown. Interestingly, in another study, plasma exosomal BACE1-AS, 51A, BC200, and BACE1 mRNA were determined but only BACE1-AS was found different in AD from controls [129], suggesting that BACE1-AS can be used as biomarkers (also if combined with imaging data of entorhinal cortex thickness).

Further studies are warranted to ascertain the relationship between lncRNAs in neuronal-derived EVs as indicative of the neuroinflammatory process leading to neurodegeneration in AD.

## 5. Discussion and Perspectives

**Figure 1:**
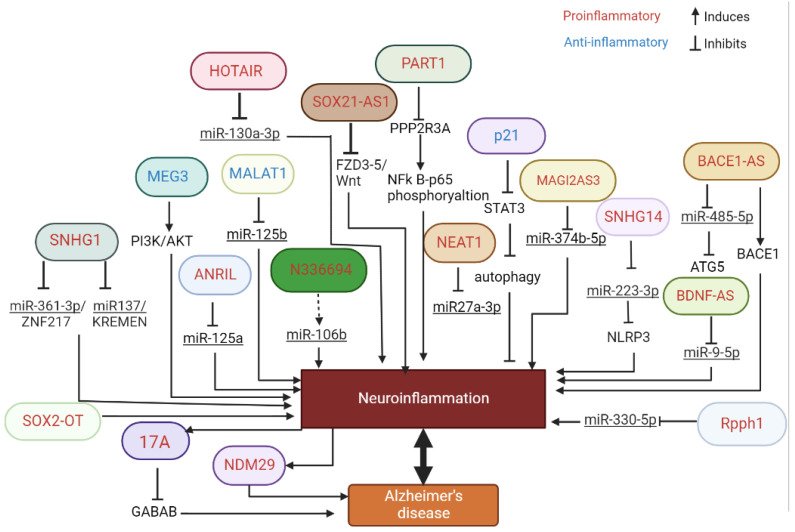
Schematic representation of the multiple pathways regulated by lncRNA involved in inflammation and AD.

In this study, we have reviewed the literature showing the association between lncRNAs and inflammation in the context of AD (Figure 1). Importantly, we were interested in the lncRNA signature associated with inflammation and AD in EV, as potential biomarkers and/or potential therapeutic targets.

The alterations in lncRNA expression signature within EVs’ cargo can be considered as disease-specific alteration, whereas neuroinflammation is a well-accepted general process underlying almost every degenerative disease. Interestingly, inflammatory processes can regulate lncRNA differently in AD and PD, confirming the lncRNA signature in different pathologies. Furthermore, lncRNA can, in turn, be regulated by inflammatory processes, perhaps in a disease-specific manner.

Given the importance of EVs in cellular communication, it is likely that the lncRNA profile alterations in damaged cells could spread the pathological situation by EVs to neighbor and distant cells [122], placing lncRNA (within EVs’ cargo) upstream of inflammation in the recipient cells, but downstream of toxic insult and inflammatory mediators in the cell of origin, very likely in a disease-specific manner.

Further studies are required to confirm if the alterations in the lncRNAs’ profile in inflammation are reflected specifically in the EVs of neural origin. Moreover, the possibility to isolate different cellular origin EVs from patients’ blood, and distinguish lncRNA signatures in them, may be a unique noninvasive strategy to understand the progression of AD and to develop effective tools for disease-risk prediction and/or potential effective treatment.

Up to date, only a few studies have been found addressing lncRNA in EVs in the context of AD, in remarkable contrast with the studies on miRNAs in EVs. Two pioneer studies have pointed to BACE1-AS1 as a biomarker of disease [125,129]. The limitations of these studies may depend on the technical question, unsolved up to date, that is to reliably isolate EVs from neural origin in blood. In addition, no inflammation marker was included in this study. Most importantly, the subjects were already diagnosed AD patients and controls, so whether BACE1-AS1 is a potential predictor of risk is still unknown. Future studies are warranted to identify lncRNAs’ signature earlier (mild cognitive impairment) to allow for early intervention.

The field of lncRNAs in AD and EVs is an emerging field that may open new routes to identify biomarkers, with potential applications for the prediction of the risk to develop the disease, and/or patients’ stratification that will afford better and effective intervention.

## Figures and Tables

**Table 1:** Long non-coding RNAs related to inflammation and Alzheimer’s disease. LncRNAs are described in alphabetical order. In red are the putative pro-inflammatory lncRNAs and in blue are the putative anti-inflammatory lncRNAs.

Lnc	Relation to Inflammation (Ref)	Regulation in AD (Ref)	Mechanism of Action (Ref)
**17A**	Upregulated by IL-1α (SH-SY5Y cells) [55]	Upregulated in the cerebral cortex (AD patients) [55]	Regulates GABA_B_ alternative splicing, leading to impaired GABA_B_ signaling [55]
**ANRIL**	Knockdown decreases inflammation, and autophagy (P12 cells) [56]	Upregulated by Aβ_1–42_ Knockdown decreases Aβ-induced apoptosis (P12 cells) [56]	Regulates miR-125a/NF-KB [56,57]
**BACE1-AS**	Knockdown decreases autophagy and alleviates neuronal injury (AD mice model) [58]	Upregulated in Serum (AD patients) Upregulated in the brain (AD mice model) [58]	Increases the stability of BACE1 mRNA miR-485-5p sponge [59] Regulates miR-214-3p/CDIP1 axis [60] Regulates miR-214-3p/ATG5 axis [58]
**BDNF-AS**	Upregulated by H_2_O_2_ exposure (SH-SY5Y cells) [61] Downregulated by lithium exposure (rat model) [61]	Upregulated in peripheral blood (AD patients) [62]	miR-9-5p sponge [61]
**HOTAIR**	Upregulated by LPS (microglial BV2 cells) [53] Inhibited by sulfasalazine (Mice model of cuprizone-induced demyelination) [63]	Downregulated in the brain by exercise (Rat model) [64]	miR-130a-3p sponge [64] Regulates miR-5p-AKT2-NF-kB axis [63]
**N336694**	Reduces inflammation (AD mouse model). Represses apoptosis (SH-SY5Y cells) [65]	Upregulated in the brain. (AD mice model) [65]	miR-106b sponge [65]
**NDM29**	Upregulated by IL-1α. (several neuroblastoma cell lines) [66]	Upregulated in the cerebral cortex (AD patients) [66]	Increases APP synthesis and actively promotes Aβ_1–42_ secretion [66]
**NEAT1**	Activates NF-κB signaling (H9c2 cells) [67]	Upregulated in the temporal cortex and hippocampus. (AD patients) [66]	miR-27a-3p sponge [68] Regulates microRNAs in AKT, TLR4, TRAF6, and NF-κB signaling pathways [69]
**PART1**	Activates NFκB-p65 signaling (endothelial cells) [70]	Increased by Aβ (endothelial cells) [70]	Upregulates PPP2R3A mRNA, responsible for NFκB-p65 phosphorylation [70]
**SNHG1**	Induce inflammation (PC12 cells) [71]	Increased by Aβ_25–35_ (SH-SY5Y cells). [72]	Regulate miR-137/ KREMEN1 [73] Regulates miR-200/ZNF217 [71]
**SNHG14**	Promotes inflammation (astrocytes from APP/PS1 mice model) [74]	Upregulated (AD patients) [75]	Regulates miR-233-3p/NLRP3 [74]
**SOX2-OT**	Promotes inflammation, apoptosis, and oxidative stress (SH-SY5Y cells) [76]	Upregulated (AD mice model) [76]	Regulates miR-942-5p/NAIF1 [76]
**SOX21-AS1**	Promotes oxidative stress (AD mice model) [77]	Upregulated (AD mice model) [77]	Upregulates FZD3-5/Wnt signaling pathway. miR-107 sponge [77]
**LincRNA-p21**	Reduces neuroinflammation (BV-2 cells) [78]	Promotes autophagy (AD mice model). Upregulated by Bilobalide (AD mice model) [78]	Inhibits STAT3 (direct binding) [78]
**NEAT2 (MALAT1)**	Increased by LPS (BV2 cells) [79])	Downregulated in the CSF (AD patients) [80]	miR-125b sponge [81] Increases miR-155the anti-inflammatory [80]
**MEG3**	Reduces Aβ_25–35_ induced inflammation (rat model) [82]	Downregulated in the hippocampus (Rat model) [82]	Inactivates PI3K/AKT pathway [82]
**Rpph1**	Induces inflammation under low glucose conditions (mesangial cells) [83]	Upregulated in the cortex (AD mice model) [84] Neuroprotector in SH-SY5Y [85]	miR-330-5p, miR326-3p, and miR122 sponge (SH-SY5Y cells) ([85], p. 21)
